# MiRNA Expression in Neuroendocrine Neoplasms of Frequent Localizations

**DOI:** 10.3390/ncrna7030038

**Published:** 2021-06-25

**Authors:** Alexandra Korotaeva, Danzan Mansorunov, Natalya Apanovich, Anna Kuzevanova, Alexander Karpukhin

**Affiliations:** Research Centre for Medical Genetics, 1 Moskvorechye St., 115522 Moscow, Russia; kor2306@mail.ru (A.K.); gah3ah@mail.ru (D.M.); apanovich2004@mail.ru (N.A.); anka.kuzevanka@yandex.ru (A.K.)

**Keywords:** miRNA, expression, neuroendocrine tumors, neuroendocrine neoplasms, biomarkers

## Abstract

Neuroendocrine neoplasms (NEN) are infrequent malignant tumors of a neuroendocrine nature that arise in various organs. They occur most frequently in the lungs, intestines, stomach and pancreas. Molecular diagnostics and prognosis of NEN development are highly relevant. The role of clinical biomarkers can be played by microRNAs (miRNAs). This work is devoted to the analysis of data on miRNA expression in NENs. For the first time, a search for specificity or a community of their functional characteristics in different types of NEN was carried out. Their properties as biomarkers were also analyzed. To date, more than 100 miRNAs have been characterized as differentially expressed and significant for the development of NEN tumors. Only about 10% of the studied miRNAs are expressed in several types of NEN; differential expression of the remaining 90% was found only in tumors of specific localizations. A significant number of miRNAs have been identified as potential biomarkers. However, only a few miRNAs have values that characterized their quality as markers. The analysis demonstrates the predominant specific expression of miRNA in each studied type of NEN. This indicates that miRNA’s functional features are predominantly influenced by the tissue in which they are formed.

## 1. Introduction

NENs are a rare family of tumors that arise from neuroendocrine cells. These cells, with their main function being to secrete hormones, are widely dispersed throughout the human body. NENs can seriously disrupt organ function [1]. Nearly 80% of patients with NENs have metastases. This is due, in particular, to difficulties in early diagnosis connected with the absence of symptoms at an early stage of the disease and the small size of tumors at such a stage. The prognosis of this disease is often poor, in addition to diagnostics problems, and the effectiveness of NEN therapy is not very effective [2]. The division of NEN tumors into subtypes not only has prognostic value, but is also important for treatment [3,4].

Difficulties with the treatment of patients with NENs enhance the desire to create methods for diagnosing and predicting the development of NENs, which would allow for individualizing the treatment of patients, as well as finding effective targets for targeted therapy.

The study of microRNA expression in NEN tumors can contribute to the solution of these questions. MicroRNAs (miRNAs) are small noncoding RNAs ~17–25 nucleotides in length, which play an important role in the regulation of gene activity at the post-transcriptional level. MiRNAs are involved in most cellular processes, including the malignancy of cells and the development of cancer [5].

MiRNAs can act as oncogenes and tumor suppressors. MiRNA expression profiles are studied for subsequent clinical use, including searching for a relationship between miRNA expression and tumor subtypes as a source of new biomarkers and targets for therapy of cancer tumors [6].

Molecular markers based mainly on the functional characteristics of genes are widely studied, and the results are summarized, for example, in [7]. Much remains to be studied and analyzed regarding miRNA. Certain aspects of miRNA expression in NENs have been reviewed in previous works [8,9,10]. In this review, we tried to describe the most important miRNAs in NEN tumors of the most common types to carry out a comparative analysis of their expression in different tissue types of tumors, which was absent before this work, and identify potential biomarkers.

## 2. Epidemiology

More than 12,000 people are diagnosed with NENs each year in the United States, and approximately 175,000 people live with this diagnosis [11]. In Russia, there are still no statistical data on the incidence of NENs [12,13].

### Classification, Prognosis and Features of NEN Disease

In 2018, WHO adopted a universal classification of all tumors from neuroendocrine cells. This was based on an expert agreement reached a year earlier [14]. The key differentiator of this new classification is the division into highly differentiated neuroendocrine tumors (previously referred to as carcinoid tumors) and poorly differentiated neuroendocrine carcinomas. 

There are difficulties in making a good NEN classification. This is due to the variety of tumors, conflicting terminology and not enough molecular characteristics. MiRNAs are often used to classify different types of cancer [15], and they can also be used to classify NENs.

Neuroendocrine tumors are most commonly found in the lungs, pancreas, stomach, small intestine and rectum. Gastroentero–pancreatic NENs account for less than 1% of gastrointestinal cancers and 7–21% of all neuroendocrine neoplasms [10]. Patients with gastric NEN can be divided into four types depending on the etiology of the tumor, pathogenesis and pathology [16]. NEN type 1 of the stomach (G1) accounts for approximately 70–80% of stomach NENs. The role of miRNA has been most studied in patients with clinical and morphological type 1 gastric NEN. These are patients with the most favorable prognoses [17]. G1 is a well-differentiated tumor associated with chronic atrophic gastritis in patients with hypergastrinemia and achlorhydria [18]. The role of miRNA in the pathogenesis of gastric NEN development against the background of hypergastrinemia has been established [17]. It was found that the expression level of miRNA-96-5p progressively increased from 1 to 3 degrees of gastric NEN. These data demonstrate the important role of miRNA as a biomarker in the classification of gastric NENs [19].

NENs of the lungs account for approximately 25% of primary lung neoplasms. They are classified into the following subtypes: typical carcinoids (well-differentiated, low grade); atypical carcinoids (well-differentiated); large cell neuroendocrine carcinomas (poorly differentiated, highly differentiated); and small cell lung cancer (poorly differentiated, high-grade) [2].

Until recently, there was a lack of specific and sensitive biomarkers for the division of NENs by subtype [20]. New data have now appeared [21,22]. Wong et al. built a classifier based on the expression of miRNA-18a and -155. The classifier made it possible to distinguish typical and atypical carcinoids from small cell and large cell neuroendocrine carcinomas with an accuracy of >90% [21]. Nanayakkara et al., using machine learning approaches, identified 17 miRNAs to distinguish 15 pathological types of NEN and subsequently constructed a multilayer classifier that correctly identifies 217 (98%) out of 221 samples. They identified common and type-specific tissue miRNA markers and built an accurate miRNA-based classifier to better understand the diversity of NENs [22]. These studies have limitations that are commonly found in the study of rare cancers and miRNAs. However, miRNAs are useful for classifying NENs and should be included in further multivariate studies of these neoplasms.

NENs are difficult to diagnose due to the lack of symptoms in the early stages of the disease. Primary neuroendocrine neoplasms can remain small for a long time and become symptomatic only after the onset of metastases. Tumors often grow slowly and do not cause discomfort to patients until they reach the terminal stage. Another feature of this type of cancer is its unpredictability. Tumors can be slow-growing or, conversely, have a high aggressiveness of growth.

For the diagnosis and treatment of NEN diseases, new biomarkers are needed that could be applied in practice in order to predict and individualize therapy. MiRNA-based therapies are also being actively studied. MiRNA drugs can be highly specific and effective. Due to their ability to regulate a wide range of genes by acting on many mRNAs, they can be effective in the fight against NENs [23].

## 3. The role of MiRNA in Neuroendocrine Neoplasms

The search for works with the expression of microRNA in NEN tumors was carried out on the PubMed, PMC, Omicsonline and Embase databases using the following keywords: miRNA, microRNA, neuroendocrine tumors, neuroendocrine neoplasms.

The miRNAs differentially expressed in NEN tumors, described in the studies found, and some of their characteristics are presented in Table 1 and discussed below.

Unlike a number of other malignant diseases, miRNA expression in patients diagnosed with NENs is not well understood. One of the reasons for this is the low prevalence of this disease. MiRNA expression among patients with NEN of the pancreas, small intestine, stomach and lung cancer have been studied [7,16,18,21,22,24,25,28,35,36,40,46,48,49,50].

In an extensive study by Nanayakkara et al., miRNA expression profiles were obtained by the small RNA sequencing approach, which allowed the authors to classify various types of NEN using original analysis. A method for NEN classification was created, and a number of other significant results were obtained [22]. For instance, the authors found that miRNA-375 expression was always increased in different types of NEN compared to tissue without NENs [22].

There is a suggestion that miRNA-375 is a universal marker of neuroendocrine cell differentiation. Increased expression of miRNA-375 is currently considered a specific marker for NENs [22]. MiR-375 can regulate the neuroendocrine differentiation of NENs of the small intestine [24].

Although expression of miRNA-7 is less common than miRNA-375, it is also often increased in NENs compared to controls without NENs [22]. It is known that miRNA-7 has an increased expression in the pituitary gland [51]. Expression of miRNA-7 in thyroid C cells [52], enteroendocrine cells [29] and pancreatic islet cells [53] suggests that this miRNA also has some degree of neuroendocrine specificity [22].

### 3.1. Gastric NENs

In the NENs of the stomach, miRNA-222 and miRNA-202 were studied. It has been shown that the level of miRNA-222 increases in the gastric mucosa and in the blood plasma against the background of hypergastrinemia. The function of miRNA-222 is to reduce the mRNA level of the cell cycle inhibitor p27KIP1 [32]. An increase in the expression level of miRNA-222 leads to the development of a tumor [32]. The action of miRNA-222 leads to a decrease in the p27 protein. MiRNA binds to the 3’-end of the *p27* mRNA [54], which leads to its degradation. It has been shown that miRNA-222-induced inhibition of the *p27* gene has an effect on tumor development [32]. MiRNA-222 has several mRNA gene targets, including *p27 kip1, p57, PUMA, PTEN, Bim* and *MMP1* [55]. MiRNA-222 also interacts with the tumor suppressor *PTEN* in gastric cancer cells [56]. This inhibits the Akt pathway and also promotes proliferation due to the suppression of the *p27* and *TP57* genes [48]. It is assumed that miRNA-222 inhibits the translation of the *VGLL4* gene and promotes YAP-TEAD activation, which also promotes tumor proliferation [28]. 

It was demonstrated that miRNA-202-3p has increased expression in gastric NENs of the first clinical morphological type [18]. One of the targets of this miRNA is the gastric tumor suppressor gene *DUSP1*. The action of miRNA-202-3p decreases the expression of this gene [18].

In one of the most recent studies [19], eight miRNAs were found that were expressed in all classes of gastric NEN (miRNA-10b-5p, miRNA-130b-3p, miRNA-192-5p, miRNA-194-5p, miRNA-210-3p, miRNA- 214-3p, miRNA-7-5p and miRNA-96-5p), but the level of their expression was different for different classes of gastric NENs. Among these miRNAs was miRNA-96-5p, the expression level of which increased with a change in the degree of differentiation of gastric NEN tumors from G1 to G3. It was also shown that miRNA-96-5p controls the expression of the *FoxO1* gene [19]. The transcription factor *FoxO1* (Forkhead box O, FOXO) is a downstream target in the IGF-1R PI3K/Akt pathway involved in several physiological and pathological processes, including cancer development. In malignant neoplasms, *FoxO1* is an important tumor suppressor gene and is inhibited in many types of tumors during their development [57]. The expression level of miRNA-96-5p is higher in grade 3 gastric NENs. Accordingly, the *FoxO1* gene has been shown to have reduced expression in grade 3 gastric NENs. *FoxO1* expression was found in tumors at G1 and G2, which is consistent with a more favorable prognosis in these patients [19]. These results demonstrate the potential value of miRNA quantification for the classification of gastric NEN subtypes.

### 3.2. Small Intestine NENs

Among the NENs of this anatomical location, a number of differentially expressed microRNAs were revealed. In particular, the expression of miRNA-204-5p, miRNA-7-5p and miRNA-375 is increased in primary tumors of the small intestine relative to normal tissue [6]. It was also shown that the expression of miRNA-1 and miRNA-143 is decreased in NENs of the small intestine and in their metastases relative to normal tissue. A decrease in the expression of miRNA-1 and miRNA-143 leads to the activation of the *FOSB* and *NUAK2* oncogenes. Expression of these genes is increased in lymph nodes and liver metastases, compared with primary NENs of the small intestine. Altered miRNA-1 expression affects the *VEGFA* gene. VEGFA levels are significantly higher in lymph nodes and liver metastases compared to primary NENs of the small intestine [6]. When the NENs of the small intestine metastasize, initially increased expression of miRNA-375 is reduced [24].

Increased expression of miRNA-182, miRNA-196a and miRNA-200a and decreased expression of miRNA-31, miRNA-129-5p and miRNA-133a were not only found in tumor tissue but also in the blood serum of patients with NENs of the small intestine [34]. Among patients with intestinal NENs, a decreased expression of miRNA-186 was found in tumor tissue, blood and stool samples compared to the control group. Along with the decreased expression of miRNA-186, an increase in *PTTG1* expression was observed in the same samples. Based on this, the authors suggested that the increased *PTTG1* expression was caused by a decrease in miRNA-186 [26]. These results indicate the possibility of non-invasive NEN diagnostics in the small intestine in the future.

Existing data also indicate some mechanisms associated with the emergence of small intestine NENs and the possibility of their further study. In addition, microRNAs participating in the development of such tumors have been identified. S.C. Li et al. showed that significant increases in miRNA-96/-182/-183/-196/-200 expression are associated with the progression of small bowel NENs [34].

Several differentially expressed miRNAs have been shown to regulate tumor cell proliferation in small intestine NENs [24]. Decreased expression of miRNA-137, miRNA-204-5p, miRNA-486-5p and miRNA-30c leads to tumor suppression. At the same time, increased expression of miRNA-21 and miRNA-1290 stimulate tumor development [24].

### 3.3. Pancreas NENs

Different levels of miRNA expression in patients with NENs and acinar (common) pancreatic tumors were demonstrated [35]. In particular, reduced expression of miRNA-155 distinguishes pancreatic NENs from acinar cell carcinomas [9,10]. MiRNA-155 interacts with mRNA of the TP53 gene, which encodes a pro-apoptotic tumor protein [9]. The expression of miRNA-103, miRNA-107, miRNA-1290, miRNA-144/451 cluster and miRNA-21 is increased. In vitro experiments showed that miRNA-144 induced the proliferation of mouse pancreatic cells and influenced Akt signaling by acting on the *PTEN* gene [9,10].

The role of several miRNAs in the development of pancreatic NENs was shown. For instance, miRNA-451 promotes tumor cell proliferation by affecting the expression of *p19* [10,33]. MiRNA-224 expression was revealed to have a significant role in the development of pancreatic NENs [38]. The direct target of this miRNA is the *PCSK9* gene. The increased expression of miRNA-224 leads to a decrease in *PCSK9* expression and an increase in glucocorticoid levels, which increases apoptosis and decreases proliferation. However, high levels of miRNA-196a stimulate the growth of pancreatic NEN tumor cells. MiRNA-196a suppresses *HOX* target genes, including *HOXB8, HOXD8* and *HOXA7* [28,58]. In addition, miRNA-196a is involved in the epithelial–mesenchymal transition, possibly through the activation of *NFκB1α* [28,59].

In the study on a mouse model with a comparison of the results with data on human cell lines, miRNA-23b and miRNA-137 were identified as participating in the progression of pancreatic NENs. The *Sorl1* gene was identified as a target for miRNA-137, but since inhibition of this gene does not lead to effects similar to miRNA-137, the genes *Kcnmb2, Hpse, Asph* and *Robo2* are considered as promising targets. The genes *ALK7, Robo2* and *P2ry* have been identified as targets of the miRNA-23b cluster. Increased expression of miRNA-137 decreases survival, leading to increased tumor growth. The miRNA-23b cluster was markedly activated during metastasis [37].

### 3.4. Lung NENs

Mairinger et al. found highly significant differential expression in lung NENs of miRNAs: let-7d, miRNA-15b, miRNA-18a, miRNA-19, miRNA-22, miRNA-29a, miRNA-29b, miRNA-29c, miRNA-335, miRNA-340, miRNA -504, miRNA-513C, miRNA-1200, miRNA-1201 and miRNA-1286 [46]. Increased expression of miRNA-375, -21, -143, -141, let-7a, let-7f, -30d and -148a in lung NENs was shown [22]. These miRNAs, according to Wong et al., account for about 30% of all miRNAs expressed in NENs of the lungs [21]. It must not be excluded that the identification in the two cited works of different microRNA sets can be due to the use of different methods of expression analysis: quantitative PCR in Mairinger et al. [46] and RNA sequencing in Wong et al. [21]. Earlier, a decreased expression of miRNA-150 and miRNA-886-3p was found in lung NENs, while the expression of miRNA-92a2 and miRNA-7 was increased [42,44,60]. Expression of miRNA-886-3p is regulated by methylation of the gene promoter encoding this miRNA and, possibly, is capable of affecting cell proliferation, migration and invasion [43]. An increased level of the expression of miRNA-21 and miRNA-34a was shown in different types of lung NEN [41]. In an investigation of miRNA-205-5p and miRNA-375-3p expression in NENs and other malignant lung tumors, it has been shown that miRNA-205-5p does not have discriminatory properties, in contrast to miRNA-375-3p [39].

Consequently, NENs in the lungs demonstrated a wide range of differentially expressed miRNAs that can be used to develop diagnostic approaches and methods for the classification of tumors. The corresponding characteristics of miRNA expression will be discussed in the following sections.

## 4. MiRNA Expression in NENs of Different Localizations: Specificity and Commonality

**Figure 1:**
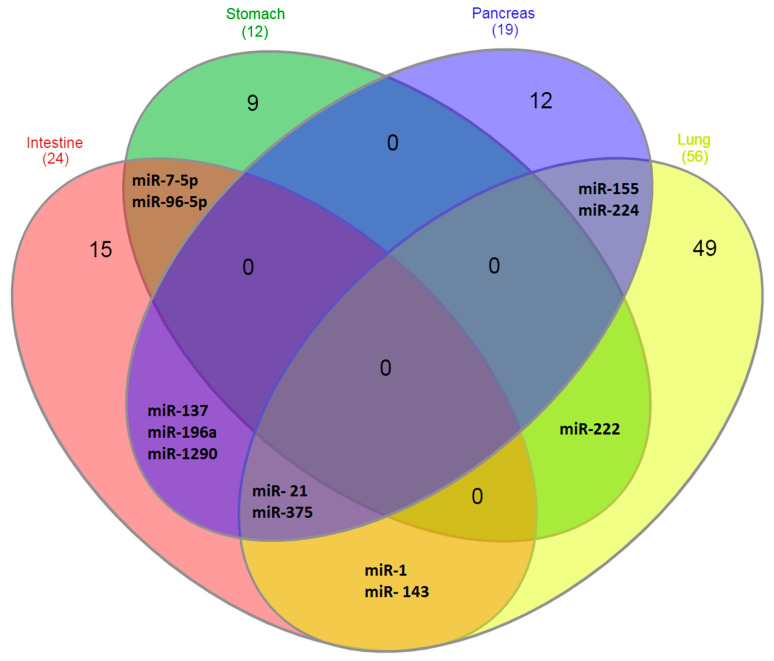
Venn diagrams. Expression of miRNA in NENs of different localizations.

Differentially expressed miRNAs in NENs localized in the intestine, stomach, pancreas and lungs, described in different works, are listed in Table 1. In the diagram in Figure 1, it is more clearly seen which miRNAs are specifically expressed in certain types of NENs. As follows from the data in Figure 1, the majority of miRNAs identified to date—about 90%—are specific for tumors of specific localizations. That is, the organ in which they are formed, and not their neuroendocrine nature, is of decisive importance for the characteristics of miRNA expression, which reflect the functional characteristics of tumors.

At the same time, some miRNAs are differentially expressed in several types of NEN. These include, first of all, miRNA-375 and miRNA-21, which are expressed in NEN tumors of the intestine, pancreas and lung [22,24,25,35]. MiRNA-7-5p and miRNA-96-5p are characteristic of both NENs of the stomach and NENs of the intestine [10,19]. MiRNA-137, miRNA-196a and miRNA-1290 are characteristic of NENs of the intestine and pancreas [8,24,28]; miRNA-1 and miRNA-143 are characteristic of NENs of the intestine and lung [6,21,39,40]; miRNA-222 is characteristics of NENs of the lung and stomach [32] and miRNA-155 and miRNA-224 are characteristic of NENs of the lung and pancreas [35,38,47]. It should be noted that the largest number of miRNAs expressed in more than one type of NEN is expressed in intestinal NENs (75% expressed in different types of NEN), followed by the lung NENs. If the largest amount of miRNA is described for lung NENs, then intestinal NENs do not stand out in this regard, which additionally indicates the reliability of the miRNA expression features identified in this section. At the same time, with the expansion of studies and the identification of additional miRNAs associated with NENs, the numerical characteristics obtained in this work can be somewhat corrected, although the main conclusion is unlikely to be changed.

In favor of the obtained conclusions, this indirectly indicates some other data. For example, when analyzing miRNA expression profiles obtained by small RNA sequencing approach of 378 samples, only nine microRNAs were expressed in samples of at least five types: miRNA-375, -21, -143, -let-7a, -26a, -7, -let-7F, -125B and -141 [22]. Interestingly, when comparing genes with deleterious variants in pancreatic NENs and intestinal NENs, only 4% of genes were common for these types of NEN [61].

## 5. MiRNA as Biomarkers of Neuroendocrine Tumors

Currently, in clinical practice, several biomarkers are used to determine the prognosis for a patient: age, tumor size, presence of distant metastases, Ki67 proliferation index and levels of biochemical markers such as chromogranin A (CgA) [62]. In addition, the Ki67 proliferation index, the number of mitoses and the histological differentiation of the tumor are used to select the treatment options that should be used as the first line of treatment [63]. Elevated serum CgA levels correlate with the size, prevalence and histopathological characteristics of NENs. It is more pronounced in large, well-differentiated and metastatic tumors. Among circulating biomarkers, CgA has been measured in several types of NEN, but its value as a prognostic biomarker is limited due to a low diagnostic sensitivity of about 60% [62]. In addition, CgA values can change in a number of other diseases [62]. These limitations indicate the need to search for new NEN biomarkers.

Such biomarkers can be miRNAs, which are a class of short noncoding RNAs that modulate gene expression. Recently, the significance of miRNAs as important regulators of oncogenesis pathways was clarified, which, due to their functional significance and low susceptibility to degradation, can act as convenient non-invasive clinical NEN biomarkers [64]. The miRNA expression may be associated with many clinical characteristics of the tumor. In particular, Arvidsson et al. [24] found a relationship between miRNA expression and the proliferation rate of neuroendocrine tumors of the small intestine. A significant decrease in the expression of miR-137 and miR-204-5p was observed in tumors with a Ki67 index above 3%. Tumor progression was also associated with significant changes in miRNA expression, for example, higher expression of miRNA-95 and miRNA-210 and lower expression of miRNA-378a-3p in metastases [29]. In rectal carcinoids, miRNA-885-5p has been identified as being associated with lymphovascular invasion. Therefore, miRNA-885-5p is proposed as a potential biomarker for predicting malignancy [28].

Wong et al. showed that the expression of miRNA-18a and -155 differs in different types of carcinoids. They also identified miRNA-17, -103 and -127 as candidate markers to distinguish typical carcinoids from atypical carcinoids and miRNA-301a, -106b and -25 as candidates for distinguishing small cell lung carcinomas from large neuroendocrine carcinoma cells [21]. Panarelli et al. created a classifier that allows distinguishing between the various types of gastroenteropancreatic neuroendocrine tumors on expression levels miRNA-615, -92b, -125b, -192, -149, -429 and -487b with an accuracy of more than 94% [25].

Not only expression in the tissue but also the level of miRNA circulating in the blood can serve as a biomarker of the state of the NEN tumor. Therefore, circulating miRNA-222 was increased in the serum of patients with hypergastrinemia, autoimmune atrophic gastritis and gastric NEN type 1 [32]. Özdirik et al. studied miRNA-29b in serum. They showed that expression levels were significantly reduced compared to healthy controls. In addition, there was a significant correlation between chromogranin A (CgA) and relative miRNA-29b levels. Serum miRNA-29b levels were independent of tumor-related factors such as proliferative activity according to Ki-67 index, tumor classification, TMN stage of malignant tumors, somatostatin receptor expression or clinical features such as functional or non-functional disease and the presence of tumor recurrence. Their data suggest a role for serum miRNA-29b levels as a previously unrecognized biomarker for the diagnosis of NENs. However, miRNA-29 does not predict tumor stage or patient outcome [65].

The miRNAs that have diagnostic or prognostic potential, characterized in this regard to date, are summarized in Table 2. The statistical characteristics of the relationship with the investigated clinical signs are also shown there.

### 5.1. Small Intestine NENs

In some works, the following miRNAs were found, the expression of which could potentially have diagnostic value: miRNA-7-5p, miRNA-182, miRNA-183 and miRNA-96-5p have increased expression in NENs of the small intestine compared to normal tissue of the small intestine. As with NENs of the small intestine, low levels of miRNA-96 expression and high levels of miRNA-133a expression were found in carcinoids of the appendix without metastases [31].

The expression of some miRNA may allow distinguishing metastases from the primary tumor, which can be associated with tumor heterogeneity. Therefore, miRNA-182, miRNA-183 and miRNA-96 have increased expression in the metastases of NENs compared to primary tumors. In addition, decreased expression of miRNA-129-5p and miRNA-133a was also found in the metastases of small intestine NENs compared with primary tumors [34,67]. However, the metastatic tumor may differ from non-metastatic tumors for the expression of miRNA. In NENs of the small intestine, an increase in miRNA-21 expression and a decrease in miRNA-150-5p in plasma were characteristic of metastatic tumors. Moreover, low levels of miRNA-21 and high levels of miRNA-150-5p were associated with a significant increase in overall survival [30]. A decrease in miRNA-375 expression was associated with reduced survival of patients with neuroendocrine tumors of the small intestine [29]. Arvidsson et al. showed that miRNA-375 could be used as a predictive biomarker for NENs of the small intestine [24].

The metastatic tumor also potential, in connection with the survival of patients, may also reflect the association of the expression of miRNA with the progression of the tumor and the grade of its malignancy. Li et al. studied samples of NENs of the small intestine at different stages of malignant neoplasm. The aim of this study was to identify a miRNA profile that may play a significant role as a novel clinical biomarker. They characterized nine miRNAs, including five (miR-96, -182, -183, -196a and -200a) whose expression is increased during tumor progression and four with decreased expression (miR-31, -129-5p, -133a and -215) [34]. To predict the malignancy of small intestine NENs, miRNA-885-5P was considered as a potential marker [28].

### 5.2. Gastric NENs

A small number of potential markers based on the miRNA expression are known for NENs of this localization. Panarelli et al. revealed increased expression of miRNA-375 in the studied samples of gastric NEN. They suggested that this miRNA is an excellent marker and potential tumor suppressor in the gastric NEN. The authors also show miRNA-7 as a possible marker of gastric NEN [25]. Cavalcanti et al. identified a diagnostic marker for staging gastric NENs. They found that the expression level of miR-96-5p increased from stages 1 to 3 of gastric NENs [19].

MiRNA-222 can be a potential prognostic marker of induced gastrin precancerous changes in the stomach [32]. Gastrin shows its effects in the stomach mainly as a result of binding to the CCK2 receptor (CCK2R) on enterochromaphphofod-like cells [9,32]. Treatment with the antagonist drugs CCK2 gastrone/receptors can lead to tumor relapse. Before taking Netaspid (CCK2R antagonist), an increase in the expression of miRNA-222 was observed in the biopsies of the gastric body [32]. After the treatment with the CCKR2 antagonist, the miRNA-222 level decreased. Based on these data, the expression of miRNA-222 was proposed as a potential biomarker of induced gastrin precancerous changes in the stomach [9,32].

### 5.3. Pancreas NENs

To search for diagnostic NEN markers, 13 miRNAs were examined to identify the pancreatic NEN marker. MiRNA-193b was activated in both the tissue and serum of pancreatic NENs compared to controls [33]. It was also found that the miRNA-144/451 and miRNA-21 cluster has increased expression compared to normal pancreatic regions [68]. In addition, the expression of miR-1290 is increased in NENs of the pancreas, which distinguishes these patients from healthy controls and people with chronic pancreatitis [34]. Increased expression of miRNA-103 and miRNA-107 and decreased expression of miRNA-155 distinguish sporadic pancreatic NENs from acinar cell carcinomas [68]. It was also found that the miRNA-144/451 and miRNA-21 cluster has increased expression compared to normal pancreatic regions. It was found that miRNA-204 is predominantly expressed in insulinomas and correlates with the immunohistochemical expression of insulin. Interestingly, miRNA-144/451 also has increased expression in insulinomas compared to other endocrine tumors [68]. Panarelli et al. showed that miR-328 expression levels in pancreatic NENs allow distinguishing between low- and intermediate-grade tumors [25].

In addition, increased expression of miRNA-1290 was found in NENs of the pancreas [34]. This miRNA can potentially distinguish these patients from the healthy control group and patients with chronic pancreatitis [34].

The expression of some miRNA can potentially serve as an indicator of the proliferative activity of the tumor, as well as the risk of metastasis. Roldo et al. reported an increased expression of miRNA-21 in pancreatic NENs, which positively correlated with the Ki-67 proliferation index and the presence of liver metastases [35]. In addition, the expression of miRNA-642 also correlated with the Ki67 index, while miRNA-210 correlated with the presence of metastases [33].

Increased expression of miRNA-3653 is also associated with an increased risk of metastasis in pancreatic NENs [36]. MiRNA-23b has been characterized as stimulating metastasis of pancreatic NENs and miRNA-137 as a stimulator of tumor growth and invasion [37]. However, given that these data were obtained primarily in a model, these miRNAs should be considered more promising for research in the search for markers rather than as potential markers.

MiRNA-196a has been identified as a prognostic factor in pancreatic NENs, as its expression is significantly associated with the tumor stage. In addition, high levels of miRNA-196a have been associated with decreased overall survival and relapse-free survival. The hazard ratio of recurrence in patients with high miRNA-196a expression was 16.267 [28]. In NENs of the pancreas, miRNA-449a performs an oncogenic function, apparently plays an important role in proliferation and may be a potential predictor of poor survival [66].

### 5.4. Lung NENs

In the NENs of this localization, when searching for markers, the expression of miRNAs was preferably studied from the point of view of tumor classification, the prediction of survival and metastasis, as well as chemoresistance. As a search for a potential diagnostic marker, the study of the discriminatory ability of some miRNAs in a recent work [39] should be noted. It was found that miR-375-3p by expression level distinguishes low-grade neuroendocrine lung tumors from non-neuroendocrine lung tumors, with 92.6% sensitivity and 90.4% specificity. Demes et al. showed that miRNAs are expressed differently in neuroendocrine tumors of the lungs with different degrees of differentiation. An increased level of miRNA 21 expression was shown to be associated with high-grade carcinomas and miRNA-34a with atypical neuroendocrine carcinomas of a low grade. If confirmed in additional studies, these miRNAs could potentially be used as practical markers for the differential diagnosis of lung cancer [41].

It was found that the expression of miRNA-22, miRNA-29a, miRNA-29b, miRNA-29c, miRNA-367, miRNA-504, miRNA-513C and miRNA-1200 negatively correlates with the degree of differentiation of lung NENs, while that of miRNA-15b, miRNA -18a, miRNA-335, miRNA-1201 positively correlates with the degree of differentiation of lung NENs [46]. The results of [40] show that the levels of miRNA-155 and miRNA-21 were significantly higher in neuroendocrine carcinomas of high malignancy compared to carcinoids. A recent study identified miRNAs capable of classifying lung NENs into subtypes. Wong et al. built a classifier based on the expression of miRNA-18a and -155. The classifier made it possible to distinguish typical and atypical carcinoids from small and large cell neuroendocrine carcinomas with an accuracy of >90%. MiRNA-17, -103 and -127 have been proposed as potential candidate markers to distinguish between typical and atypical carcinoids. MiRNA-301a, -106b and -25 can help to distinguish small-cell from large-cell neuroendocrine carcinomas [21]. In a search for prognostically significant markers, Ranade et al. investigated the expression of 880 human miRNA in small-cell lung cancer samples. They found an inverse correlation of miRNA-92a2 expression levels with patient survival [42]. In studies of 924 miRNAs, a relationship was found between low expression of miRNA-150 and miRNA-886-3p and a poor prognosis of patient survival [60]. It should be noted that the prognostic value of miR-886-3p expression was also found in the previous work [43]. A negative correlation was found between survival and the expression levels of three miRNAs (miRNA-192, miRNA-200c and miRNA-205) [45]. MicroRNA let-7d, miRNA-19, miRNA-576-5p, miRNA-340 and miRNA-1286 are also associated with the survival of NEN patients [46].

When examining the expression of a panel of seven miRNAs (miRNA-21, miRNA-29b, miRNA-34a/b/c, miRNA-155 and let-7a), no association was found between miRNA expression levels and survival or drug resistance [69]. However, the expression levels of miRNA-21 correlated with metastases in the lymph nodes, indicating a prognostic role of this miRNA in NENs of the lungs [40]. At the same time, when using a panel of 1145 miRNAs for examining 47 NEN samples of the lungs, no association was found between miRNA expression and patient survival [47]. In another publication, increased expression of miRNA-21 and decreased expression of miRNA-409-3p, miRNA-409-5p and miRNA-431-5p correlated with the presence of lymph node metastases and with overall survival [50].

The levels of expression of miRNA-92a-2, miRNA-147 and miRNA-574-5p were found to be associated with chemoresistance [42]. The expression levels of miRNA-7 in lung NENs negatively correlated with chemoresistance [44]. This is perhaps due to the effect on MRP1/ABCC1 [44].

Thus, a fairly wide spectrum of miRNAs with diagnostic and prognostic potential is already known. However, most of them are poorly characterized as biomarkers. In this regard, the works of Detassis and Wong should be highlighted, in which the sensitivity and specificity were determined, which amounted to about 90% [21,39]. Nanayakkara et al. showed that the expression of miRNA-375 and miRNA-7 in NENs of different types is 10 times higher in comparison with normal tissue, and developed an original approach for NEN classification [22]. Panarelli et al. reached accuracy in the gastroenteropancreatic neuroendocrine tumors classification of 94–98% [25]. We can also note the data of Bowden, Demes and Lee, who showed a high level of significance of the relationship between increased expression of miRNA-21 (*p* < 0.0001) with increased malignancy, the appearance of metastases and a significant decrease in overall survival [30,40,41]. As shown by Arvidson et al., there is a highly significant correlation between the suppression of miRNA-375 in tumor metastases and shorter patient survival. According to qRT-PCR data, the expression of this miRNA in NENs of the small intestine is 17 times higher than in the normal mucous membrane of the small intestine [24].

## 6. Conclusions

As follows from the analysis, considerable information has already been accumulated on the expression of miRNA in various types of NEN, its relationship with the development of NEN tumors and, to a somewhat lesser extent, the mechanisms of its action. For the first time, we have carried out a comparative study, based on the data available in the literature, of the distribution of differentially expressed miRNAs in NENs developing in various organs. The predominant specificity of miRNA expression in NEN tumors of a certain localization was revealed, although about 10% of the described miRNAs are expressed in several types of NEN. These results indicate in favor of a significant influence of the tissue of the localization organ on the functional features of NENs, with a certain contribution from their neuroendocrine origin.

A significant number of microRNAs are known to be potentially capable of performing the functions of biomarkers. For several miRNAs, important characteristics for biomarkers such as sensitivity and specificity were determined, amounting to about 90%. It should be noted that such miRNAs can be used not only as tissue biomarkers but, given their high stability, also for non-invasive analysis. Therefore, there are grounds for an optimistic view of the possibility of NEN markers appearing in clinical practice in the medium term.

At the same time, further studies are required to identify the spectrum of reliable diagnostic and prognostic markers of NENs, as well as to verify the already available results. In particular, it should be noted that there have been limited studies on the microRNA expression in gastric NENs. As a result, the number of potential biomarkers of NENs in this localization is very small.

## Figures and Tables

**Table 1:** Differentially expressed miRNAs at main NEN localizations.

Localization of NEN	Increased Expression	Decreased Expression	Citation
Small intestine	miRNA-375		[24]
	miRNA-375 miRNA-21 miRNA-143		[25]
	miRNA-204-5p miRNA-7-5p	miRNA-1 miRNA-143	[6]
		miRNA-186	[26]
	miRNA-133a		[27]
	miRNA-182 miRNA-196a miRNA-200a	miRNA-31 miRNA-129-5p miRNA-133a	[28]
	miRNA-21 miRNA-1290	miRNA-137 miRNA-204-5p miRNA-486-5p miRNA-30c	[24]
	miRNA-95 miRNA-210	miRNA-378a-3p	[29]
		miRNA-150-5p	[30]
	miRNA-885-5p		[28]
	miRNA-7-5p miRNA-182 miRNA-183 miRNA-96-5p	miRNA-129-5p miRNA-133a	[10]
Gastric	miRNA-10b-5p miRNA-130b-3p miRNA-192-5p miRNA-194-5p miRNA-210-3p miRNA-214-3p miRNA-7-5p miRNA-96-5p		[19]
	miRNA-96 miRNA-133		[31]
	miRNA-222 miRNA-202		[32]
Pancreas	miRNA-19b miRNA-146b	miRNA-720	[33]
	miRNA-103 miRNA-107 miRNA-1290 miRNA-144/451 miRNA-21	miRNA-155	[9,10,34]
	miRNA-21 miRNA-375 miRNA-204 miRNA-103	miRNA-155	[35]
	miRNA-196a		[8,28]
	miRNA-3653	miRNA-4417 miRNA-574-3p miRNA-664b-3p	[36]
	miRNA-23b miRNA-137		[37]
	miRNA-224 *		[38]
Lungs	miRNA-18a miRNA-155 miRNA-375 miRNA-21 miRNA-143 miRNA 141 let-7a let-7f miRNA-30d miRNA-148a		[21,39,40]
		miRNA-150 miRNA-886-3p	[38]
	miRNA-92a2 miRNA-7	miRNA-150 miRNA-886-3p	[8,10]
	miRNA-34a miRNA-21		[41]
	miRNA-92a-2 miRNA-147 miRNA-574-5p		[42]
		miRNA-150 miRNA-886-3p	[43]
	miRNA-7		[44]
	miRNA-192 miRNA-200c miRNA-205		[45]
		miRNA-409-3p miRNA-409-5p miRNA-431-5p	[8,10]
	let-7d miRNA-19 miRNA-576-5p miRNA-340 miRNA-1286		[46]
	miRNA-129 miRNA-323-3p miRNA-487b miRNA-410 miRNA-369-3p miRNA-376a miRNA-432 miRNA-129-3p miRNA-409-3p miRNA-494 miRNA-376a: 9-1 miRNA-136 miRNA-370 miRNA-127-3p miRNA-154 miRNA-376a	miRNA-203 miRNA-224 miRNA-155 miRNA-302d miRNA-34b miRNA-181b miRNA-193a-5p miRNA-34b miRNA-222 miRNA-30a-3p miRNA-938 miRNA-218 miRNA-511 miRNA-34c-3p miRNA-10a miRNA-146a miRNA-1	[47]

*—The expression level of miRNA-224 depends on the properties of the tumor.

**Table 2:** MicroRNAs as potential NEN biomarkers.

miRNA	Characteristic miRNA as Candidates for NEN Markers	Methods	Citation
	**NENs of different anatomical sites**		
miRNA-375, miRNA-7	Increased expression levels in tumor tissue compared to control	MiRNA sequencing and data mining	[22]
	**Intestines**		
miRNA-375	Increased expression in tumor biopsies compared to normal mucosa (*p* = 1.0 × 10^−19^). Can be considered as a prognostic marker. Highly expressed patients with liver metastases had significantly better survival (*p* = 0.016)	qRT- PCR	[24]
miRNA-1, miRNA-143	Decreased expression associated with tumor progression (*p* < 0.05, *p* < 0.001)	qRT-PCR	[6]
miRNA-31, miRNA-129-5p, miRNA-133a, miRNA-215	Decreased expression is associated with tumor progression (*p* < 0.05)	qRT-PCR	[34]
miRNA-96, miRNA-182, miRNA-183, miRNA-196a miRNA-200a	Increased expression associated with tumor progression (*p* < 0.05)	qRT-PCR	[34]
miRNA-21-5p miRNA-22-3p	Increased expression is associated with metastases (*p* < 0.0001) and decreased survival (OR = 0.47, 95% CI 0.27–0.82)	miRNA sequencing	[30]
miRNA-150-5p	Decreased expression is associated with the appearance of metastases (*p* = 0.027)	miRNA sequencing	[30]
miRNA-7-5p	Increased expression in tumor biopsies compared to normal mucosa (*p* = 4.2 × 10^−19^)	Chip hybridization	[24]
miRNA-615, miRNA-92b	Increased midgut expression compared to non-midgut samples (*p* < 0.01)	miRNA sequencing and qRT-PCR	[25]
miRNA125b, miRNA-192, miRNA-149	Expression is significantly lower in iliac than in appendicular NENs (*p* < 0.01)	miRNA sequencing and qRT-PCR	[25]
miRNA-186	Expression decreased in tumor (*p* < 0.05)	RT-PCR	[26]
	**Gastrointestinal tract**		
miRNA-96	Increased expression in liver metastases compared with primary NENs (*p* < 0.05)	qRT-PCR	[31]
miRNA-133a	Decreased expression in liver metastases compared with primary NENs (*p* < 0.05)	qRT-PCR	[31]
miRNA-375	Increased expression in all samples	miRNA sequencing and qRT-PCR	[25]
miRNA-202-3p	Expression increased in tumor tissue compared to normal tissue (*p* = 0.014)	RT-PCR	[18]
miRNA-429	Expression is significantly higher in rectal NENs compared to pancreatic ones (*p* < 0.01)	miRNA sequencing and qRT-PCR	[25]
miRNA-96-5p	Increased expression from G1 to G3 (*p* < 0.05)	RT-PCR	[19]
	**Pancreas**		
miRNA449a	Important role in proliferation and may be a potential predictor of poor survival	Microarrays	[66]
miRNA-196a	Increased expression is associated with decreased overall survival (*p* = 0.046). High expression indicates poor prognosis after pancreatic NEN resection. Recurrence HR = 16.26	Nanostring nCounter Analysis and qRT-PCR	[28]
miRNA-137	Reduces survival, leading to increased tumor growth	RNA sequencing	[37]
miRNA-224	Increased expression increases apoptosis and decreases proliferation (*p* < 0.05)	RT-PCR	[38]
miRNA-23b	Increased expression during metastasing	RNA sequencing	[37]
miRNA-3653	Increased expression associated with risk of metastasis (*p* < 0.05)	MiRNA microarray	[36]
miRNA-193b	An increase in expression with NENs was found not only in tissue but also in serum (*p* < 0.05)	RT-PCR	[33]
	**Lungs**		
miRNA-92a-2	Increased expression is associated with chemoresistance (*p* = 0.010) and decreased survival (*p* = 0.007)	RT-PCR	[42]
miRNA-147	Increased expression associated with chemoresistance (*p* = 0.018)	RT-PCR	[42]
miRNA-574-5p	Increased expression associated with chemoresistance (*p* = 0.039)	RT-PCR	[42]
let-7d, miRNA-19, miRNA576-5p, miRNA-340 * miRNA-1286	High expression levels are associated with survival (*p* < 0.05)	Q-PCR	[46]
miRNA-21	An increase in the level of expression correlated with an increase in the grade of malignancy (*p* = 0.00033).	Q-PCR	[41]
miRNA-34a	High expression levels associated with atypical carcinoids (*p* = 0.010)	Q-PCR	[41]
miRNA-18a miRNA -155	Increased expression allows low-grade carcinoids to be distinguished from high-grade carcinoids with a high degree of accuracy (>90%). Diagnostic marker	Sequencing	[21]
miRNA-17, miRNA-103, miRNA-127	Candidate markers for distinguishing between typical and atypical carcinoids. Accuracy 93%	Sequencing	[21]
miRNA-301a, miRNA-106b miRNA-25	Candidate markers for distinguishing small cell lung carcinoma and large cell neuroendocrine carcinoma. Accuracy 100%	Sequencing	[21]
miRNA-375-3p	Distinguishes low-grade neuroendocrine lung tumors from non-neuroendocrine lung tumors with 92.6% of sensitivity and 90.4% of specificity	qRT-PCR	[39]
miRNA-21, miRNA-155	Increased expression in high-grade tumors compared to carcinoid tumors (each *p* < 0.001). The expression level of miR-21 in carcinoid tumors with metastases to lymph nodes is higher than in carcinoid tumors without metastases to lymph nodes (*p* = 0.010)	qRT-PCR	[40]

*—The expression level of miRNA-340 depends on the properties of the tumor.
